# Nystagmus in children with primary brain tumours in Denmark between 2007 and 2017

**DOI:** 10.1038/s41433-023-02771-x

**Published:** 2023-10-10

**Authors:** Jacqueline Gremaud Rosenberg, Kamilla Nissen, Steffen Heegaard, Suganiah Ragunathan, Kjeld Schmiegelow, René Mathiasen, Sarah Linea von Holstein

**Affiliations:** 1 Department of Pediatrics and Adolescent Medicine, Copenhagen University Hospital, Rigshospitalet, Denmark; 2 Department of Ophthalmology, Copenhagen University Hospital, Rigshospitalet-Glostrup, Denmark; 3 Department of Pathology, Copenhagen University Hospital, Rigshospitalet, Denmark; 4 Institute of Clinical Medicine, Faculty of Medicine, University of Copenhagen, Copenhagen, Denmark; 5 Department of Ophthalmology, Aalborg University Hospital, Aalborg, Denmark

**Keywords:** Eye manifestations, Epidemiology, Cancer

## Abstract

**Background:**

The aim of the study was to evaluate the prevalence, clinical characteristics, and diagnostic importance of nystagmus in children with brain tumours.

**Methods:**

A nation-wide retrospective review of all children diagnosed with a brain tumour between January the 1st, 2007 and December 31st, 2017, in Denmark. Data is based on information from the Danish Childhood Cancer Registry, hospital records from paediatric- and ophthalmological departments, and records from private ophthalmologists.

**Results:**

Nystagmus was observed in 13.7% (60/437) of children with a brain tumour. In 50/60 children (83.3%) nystagmus was an incidental finding at the clinical examination and only in 10/60 children (16,7%) were nystagmus noticed by patient/caregivers prior to the clinical examination. In 38/60 children nystagmus was observed before the brain tumour diagnosis, most often (16/38, 42%) the same day as the diagnosis was made. In 22/60 children nystagmus was found after the brain tumour diagnosis (prior to any treatment) with a median of four days (range 0-47) after the brain tumour diagnosis. Nystagmus was most commonly binocular (56/60, 93.3%) and gaze-evoked (43/60, 71.7%). The median number of additional symptoms and/or clinical findings was five (range 0–11).

**Conclusion:**

Nystagmus is frequent in children with brain tumours and is typically accompanied by other symptoms and clinical signs. However, nystagmus is often first recognized by the ophthalmologist late in the time course. Therefore, raising awareness of the importance of looking for nystagmus in children with unspecific neurological symptoms might contribute to increased suspicion of brain tumour and thereby faster diagnosis.

## Introduction

Brain tumours are the most common solid tumours in paediatric patients and the second leading cause of death in children due to neoplasms in European countries [1]. A larger number of survivors are left with neurological disabilities and visual impairments [2]. To minimize sequelae from the tumour and to initiate less heavy treatment early diagnosis is important [3]. In many countries including Denmark diagnostic delay have been identified [4, 5] and this calls for a closer look at the onset symptoms and disease course until diagnosis. The Brain Pathways guideline, an evidence-based guideline to assist healthcare professionals in diagnosing brain tumours in children, emphasizes that new-onset nystagmus in children requires CNS imaging even in absence of other symptoms according to the high probability of an underlying CNS lesion [3]. Multiple studies either investigating brain tumour symptoms in children or nystagmus in children confirms this [4, 6–11]. Nystagmus is defined as involuntary, rhythmic, and repetitive oscillations of one or both eyes (i.e., monocular or binocular) and can occur in primary gaze position or in eccentric position of gaze, i.e., gaze-evoked nystagmus [10]. Nystagmus may occur by a disturbance in one of the three control mechanism for maintaining steady gaze: visual fixation, the vestibulo-ocular reflexes, and the neural integrator for horizontal gaze-holding, reflecting a lesion of the visual pathway, the brainstem, or cerebellum [12, 13]. Nystagmus is classified as infantile nystagmus if it appears within the first three to six months of life and as acquired nystagmus if it appears after [14]. Infantile nystagmus may also be caused by a brain tumour, but other causes such as congenital idiopathic nystagmus (also known as infantile nystagmus syndrome) is much more common [14]. Regardless of age, there are many different causes of nystagmus in addition to brain tumours including ocular diseases, idiopathic, migraine, and vestibular diseases [7, 14]. Choosing the correct investigations in the optimal priority are important to avoid unnecessary investigations. This is particular important in infants where investigations requiring general anaesthesia should be considered carefully because of potential complications for the infant.

The purpose of this nation-wide retrospective study was accordingly, to investigate the occurrence of nystagmus in children diagnosed with brain tumours with focus on the clinical characteristics and the importance of nystagmus in the diagnostic process.

## Materials and methods

This study is a nationwide retrospective review of children (age 0–18 years) with nystagmus as an onset symptom of a primary brain tumour diagnosed between January 1st, 2007 until December 31st, 2017 in Denmark.

We identified children with brain tumours using the Danish Childhood Cancer Registry (DCCR). DCCR is a nationwide clinical quality database of all children diagnosed with cancer from where we collected information on diagnosis, age- and date of diagnosis, histological diagnosis, location of tumour, mortality, and treatment [15]. We collected hospital records from paediatric and ophthalmological departments in the period of two years prior to diagnosis of a brain tumour in all children. Additional visits at private ophthalmologists in the period of two years before the diagnosis was identified using the Danish National Health Insurance Service and relevant files were collected and reviewed.

All symptoms and clinical findings leading to the diagnosis were registered. All cases describing abnormal eye movement were reviewed and only those with nystagmus confirmed by an objective examination were included. Children where the nystagmus was registered after diagnosis but prior to treatment were also included whereas children with nystagmus observed after initiating any treatment were excluded. When calculating the time from nystagmus to diagnosis, only the children with nystagmus found before the diagnosis were included. We registered if the nystagmus was binocular, or monocular, the direction of oscillations and the position of gaze with nystagmus. We defined the initial symptom to be the earliest occurring symptom. Self-observed nystagmus was defined as abnormal eye movements observed by the patient/caregivers as opposed to cases where the nystagmus was an incidental clinical finding.

Based on the Brain Pathways Guideline [3] we divided the children into three age groups (0–5 years, 6–11 years, 12–18 years) in order to detect any difference in occurrence of symptoms due to age.

### Statistics

Fisher’s exact test was used to assess any statistical association between nystagmus and various clinical and tumour variables and between symptoms and age [16]. Statistical significance was defined as a P value less than 0.05.

Kaplan-Meier method was used to estimate the survival curve, to detect the five-year overall survival. End of follow-up was 1st of May 2019. This statistical analysis was conducted in RStudio (v.3.6.3).

The study was approved by the Danish Patient Safety Authority (3-3013-2800/1) and the Danish Data Protection Agency (VD-2019-84). The study was evaluated by the Regional Research Ethical Committee of the Capitol Region, Copenhagen, Denmark and deemed not to require ethics approval. The study adhered to the Declaration of Helsinki.

## Results

A total of 437 children were diagnosed with a primary brain tumour during 2007–2017. In 94 children, complaints of abnormal eye movement and/or nystagmus at the clinical examination were described in the medical records. In total, we excluded 34 children from the study because nystagmus was first present after treatment was initiated (*n* = 23), nystagmus could not be confirmed at the clinical examination (*n* = 8), and the brain lesion was not a neoplastic tumour (*n* = 3). This resulted in a total of 60 children (13.7%) with nystagmus included in the study (Tables 1–3).

**Table 1:** Clinical characteristics of children with nystagmus and a brain tumour (*n* = 60).

Sex, no. (%)	
Female	24 (40)
Male	36 (60)
**Age at diagnosis in years, no. (%)**	
0–5	15 (25)
6–11	25 (41.7)
12–18	20 (33.3)
**Nystagmus observed, no. (%)**	
Before tumour diagnosis	38 (63.3)
After tumour diagnosis	22 (36.7)
**Tumour pathology, no. (%)**	
Low-grade	31 (51.7)
Pilocytic astrocytoma	18 (30)
Optic nerve glioma	6 (10)
Ependymoma	2 (3.3)
DNET	2 (3.3)
Diffuse astrocytoma	1 (1.7)
Meningioma	1 (1.7)
Schwannoma	1 (1.7)
High-grade	27 (45)
Medulloblastoma	7 (11.7)
DIPG	7 (11.7)
Anaplastic glioma	6 (10)
ATRT	2 (3.3)
Glioblastoma	2 (3.3)
Germinoma	1 (1.7)
NGGCT	1 (1.7)
PNET	1 (1.7)
Unspecified	2 (3.3)
**Tumour location, no. (%)**	
Supratentorial	18 (30)
Cerebral hemisphere	4 (7)
Supratentorial central	5 (8)
Hypothalamus	1 (2)
Optic nerve	2 (3)
Chiasma	3 (5)
Pineal gland	3 (5)
Infratentorial	42 (70)
Cerebellum	29 (48)
Brainstem	12 (20)
Unspecified	1 (2)
**Survival status at end of follow-up, no. (%)**^+^	
Alive	42 (70)
Deceased	18 (30)
**Five-year overall survival, (%)**	69
Time from initial symptom to diagnosis (median), days (range)*	62.5 (0–557)

^+^Assessed by 1st of May 2019.

^*^This include the whole sample (*n* = 60)

*DNET* Dysembryoplastic neuroepithelial tumour, *DIPG* Diffuse Intrinsic Pontine Glioma, *ATRT* Atypical Teratoid Rhabdoid Tumour, *NGGCT* non-germinoma germ cell tumour, *PNET* Primitive Neuro-Ectodermal Tumours.

**Table 2:** Nystagmus characteristics in relation to tumour location and mode of observation (self-reported/clinical finding) in children with brain tumours in Denmark.

	Total,	Self-reported,	Clinical finding,	*P*	Supra-tentorial,	Infra-tentorial,	*P*
	*n* (%)	*n* (%)	*n* (%)		*n* (%)	*n* (%)	
**Monocular**	4 (6.7)	2 (20)	2 (4)		3 (16.7)	1 (2.4)	
**Binocular**	56 (93.3)	8 (80)	48 (96)		15 (83.3)	41 (97.6)	
				0.13			0.77
**Total**	60 (100)	10 (16.6)	50 (83.3)		18 (30)	42 (70)	
**Primary position**	15 (25)	7 (70)	8 (16)		8 (44.4)	7 (16.7)	
**Gaze-evoked**	43 (71.7)	2 (20)	41 (82)		8 (44.4)	35 (83.3)	
				0.0006*			0.017*
**Convergence**	2 (0.3)	1(0.2)	1(0.2)		2(0.3)		

*P*-values reflect Fischer’s exact test. *Highlights significant difference.

**Table 3:** Frequency of ophthalmic symptoms and clinical findings divided by age in children with brain tumours and nystagmus.

	Total, *n* (%)	0–5 y, *n* (%)	6–11 y, n (%)	12–18 y, *n* (%)	*P*
	(*n* = 60)	(*n* = 15)	(*n* = 25)	(*n* = 20)	
**Symptoms**					
Diplopia	17 (28.3)	1 (6.7)	9 (36)	7 (35)	0.009*
Strabismus	12 (20)	4 (26.6)	4 (16)	4 (20)	0.66
Reduced/Blurred vision	10 (16.7)	1 (6.7)	4 (16)	5 (25)	0.34
Abnormal eye movements	10 (16.7)	7 (46.7)	0 (0)	3 (15)	<0.001*
**Clinical findings**					
Papilledema	16 (26.7)	2 (13.3)	5 (20)	9 (45)	0.088
Oculomotor abnormalities	15 (25)	1 (6.7)	7 (28)	7 (35)	0.16
Pupillary abnormalities	9 (15)	3 (20)	1 (4)	5 (25)	0.12
Optic atrophy	6 (10)	4 (26.7)	1 (4)	1 (5)	0.067
Retinal abnormalities	4 (6.7)	2 (13.3)	2 (8)	0 (0)	0.28

*P*-values reflect Fischer’s exact test.

*Highlights significant difference.

In 50/60 (83.3%) cases nystagmus was an incidental finding in children where other symptoms were primary complaints. In 10/60 (16.7%) children abnormal eye movements were observed by the patient or the caregivers (self-observed nystagmus) and then confirmed at the clinical examination. All patients (100%) were seen by a paediatrician and 48/60 (80%) were evaluated by an ophthalmologist. Nystagmus was identified by the paediatrician in 31% and by an ophthalmologist in 69% of cases. Median time from initial symptom to brain tumour diagnosis of all children with nystagmus was 62.5 days (range 0–557).

### Timing of nystagmus

**Fig. 1:**
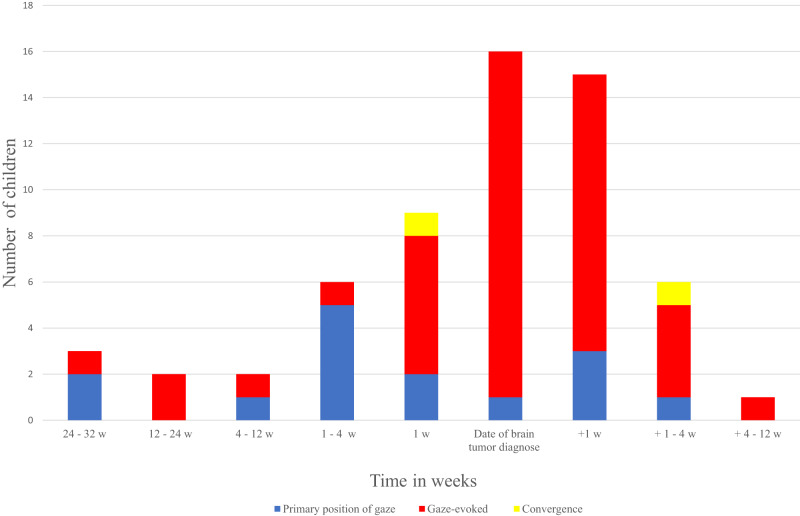
Timing of nystagmus. The y-axis describes the number of children divided by the direction of nystagmus (primary position, gazed-evoked or convergence) and the x-axis shows the timing of nystagmus (observed/identified) in relation to time of diagnosis.

In 38/60 cases nystagmus was observed before the diagnosis of a brain tumour and in 16 of these the observation was the same day as the brain tumour was diagnosed (Fig. 1). In 22 cases the ophthalmic examination describing the nystagmus was performed after the diagnosis, but prior to any treatment. The median time in this group was four days (range 0–47) after the brain tumour diagnosis. Whether nystagmus was found before or after the diagnosis did not depend on the nystagmus being in primary position or gaze-evoked (*p* = 0.53). Of the cases with self-observed nystagmus (*n* = 10/60, 16.6%), median time from nystagmus was observed to brain tumour diagnosis was 37.5 days (range 3–214). In the same cases median time from initial symptom to brain tumour diagnosis was 70.5 days (11–395 days). Nystagmus was the initial symptom in 4/60 (6.7%) children.

### Additional symptoms and clinical findings

One patient had nystagmus/abnormal eye movements as the only presenting symptom whereas all other patients had additional complaints including ophthalmic and non-ophthalmic symptoms and/or clinical findings. The mean number of additional symptoms and/or signs was five (range 0–11) (Tables 3, 4).

**Table 4:** Frequency of non-ophthalmic tumour signs divided by age in children with brain tumours and nystagmus.

	Total, *n* (%)	0–5 y, *n* (%)	6–11 y, *n* (%)	12–18 y, *n* (%)	*P*
	(*n* = 60)	(*n* = 15)	(*n* = 25)	(*n* = 20)	
**Tumour signs**					
Headache	39 (65)	5 (33.3)	19 (76)	15 (75)	0.017*
Imbalance	34 (56.6)	5 (33.3)	17 (68)	12 (60)	0.11
Vomiting	29 (48.3)	6 (40)	15 (60)	8 (40)	0.35
Nausea	21 (35)	3 (20)	10 (40)	8 (40)	0.38
Behavioural change	18 (30)	6 (40)	7 (28)	5 (25)	0.058
Focal neurological deficits	16 (26.7)	1 (6.7)	10 (40)	5 (25)	0.058
Loss of appetite	14 (23.3)	5 (33.3)	6 (24)	3 (15)	0.47
Weight loss	12 (20)	2 (13.3)	7 (28)	3 (15)	0.54
Abnormal head posture	8 (13.3)	4 (26.7)	3 (12)	1 (5)	0.24
Ataxia	8 (13.3)	2 (13.3)	4 (16)	2 (10)	0.89
Hearing loss	8 (13.3)	1 (6.7)	2 (8)	5 (25)	0.18
Cranial nerve palsy	8 (13.3)	1 (6.7)	5 (20)	2 (10)	0.45
Seizures	4 (6.7)	1 (6.7)	1 (4)	2 (10)	0.82
Macrocephaly	2 (3.3)	2 (13.3)	0 (0)	0 (0)	0.059
Endocrine involvement	2 (3.3)	0 (0)	1 (4)	1 (5)	1

*P*-values reflect Fischer’s exact test. *Highlights significant difference.

### Nystagmus characteristics

Nystagmus was reported as binocular in 56/60 cases (93.3%) (Table 2*)*. It was either gaze-evoked (*n* = 43, 71.7%), observed in primary position (*n* = 15, 25%), or present at convergence (*n* = 2, 3.3%). The direction of nystagmus was horizontal in 11/60 (18.3%), torsional in 3/60 (5%), vertical in 1/60 (1.6%), and combined in 9/60 (15%). In 36/60 (60%) the direction was not reported. Gaze-evoked nystagmus was more often an incidental finding as opposed to being self-observed (41 vs. 2, *p* = 0.0006), whereas nystagmus in primary position was equally frequent an incidental finding as self-observed (7 vs. 8). Whether nystagmus was binocular or monocular did not correlate with location (*p* = 0.77) and did not correlate with how nystagmus was discovered (i.e., self-observed or incidental finding, *p* = 0.13).

Of the self-observed cases, the age distribution was bimodal (Table 3). In the youngest children (0-5 years) nystagmus in primary gaze position was the only form reported. In the oldest age group (12–18 years), nystagmus was reported solely as gaze-evoked or present at convergence.

### Tumour location and follow-up

The brain tumours were more frequently located infratentorial (42/50, 70%) than supratentorial (18/50, 30%) and most frequently found in the cerebellum/fourth ventricle (29/60, 48.3%) or the brainstem (12/20, 20%) (Table 1). Tumours affecting the visual pathway i.e., optic gliomas (involving the optic nerve, chiasm, and hypothalamus) were only observed in the youngest children ( < 5 years). Patients with gaze-evoked nystagmus were more likely to have an infratentorial tumour than a supratentorial tumour (8 vs. 35, *p* = 0.017) (Table 2).

Five-year overall survival among children with nystagmus was 69%.

## Discussion

In this nationwide retrospective study of children with CNS-tumours, we describe the reported cases of nystagmus in children diagnosed with a brain tumour during an eleven-year period in Denmark. We find that nystagmus is a common tumour sign, but that it often goes unnoticed by the patients, caregivers and paediatricians and is first discovered late in the time course at the ophthalmological examination.

We found that eye movements were not always assessed by the paediatricians and in 20% the paediatricians had not described the nystagmus initially. The nystagmus was first reported at the time of an ophthalmological examination and because not all 437 children underwent ophthalmological examination our prevalence of nystagmus at 13.7% (*n* = 60/ 437) may be underestimated. In the existing literature, the prevalence of nystagmus in children with brain tumours vary substantially between studies from different medical specialists [4, 8, 11] confirming this notion. Thus, two studies based on examinations by ophthalmologists found a prevalence of nystagmus in children with brain tumours of 24% [11] and 40% [8]. While a study by paediatricians and neurosurgeons reported abnormal eye movements in only 3% [4]. The difference in these numbers may illustrate difficulties in recognizing subtle forms of nystagmus as well as lack of examination of eye movements by physicians in general - which is necessary to detect gaze-evoked forms. The inclusion of records by both paediatricians and ophthalmologists in the present study attempts to minimize the risk of further underestimating the prevalence.

Nystagmus was in just one child the only present symptom prior to diagnosis. In all other cases additional ophthalmic and non-ophthalmic symptoms where present and often the primary reason for seeking medical evaluation. These findings raise two important notions. One that the children had other neurological or ophthalmological signs in addition to nystagmus that help raise the suspicion of a brain tumour. This is important when evaluating infants with infantile nystagmus where a thorough examination may reveal other causes of the nystagmus and consequently exclude the need for an MRI. The other notion is that children with unspecific symptoms that might mimic other more common childhood diseases, should undergo a thorough neurological and ophthalmological examination as this will in many cases reveal whom to refer for MRI. The finding of nystagmus would be a clear indication that further investigations including MRI are needed [3, 4, 6–11].

In the present study nystagmus was in many cases found the same day as the brain tumour was given (*n* = 16/38, Fig. 1) and the nystagmus along with other symptoms were the reason for performing MRI. Taken together our study emphasizes the importance of testing oculomotor function in children with unspecific symptoms. Testing of oculomotor function is costless, easy, and isn’t associated with discomfort for the patient. Consequently, reminding physicians about testing and looking for nystagmus including gaze-evoked forms could perhaps speed up the diagnostic process for some children.

In the UK many children had a prolonged diagnostic interval (time from symptom onset to diagnosis) of 14.4 weeks [3]. After The Brain Pathways Guideline was publicized throughout a public and professional awareness campaign called The HeadSmart: Be Brain Tumour Aware campaign, the diagnostic interval was reduced to 6.7 weeks in 2013 [3]. In our study population the median diagnostic interval was 62.5 days (8.9 weeks). Children with self-observed nystagmus were not diagnosed earlier than the rest of the group (median time from initial symptom 70.5 days and from observation of nystagmus 37.5 days). Self-observed nystagmus was significantly associated with the youngest children and to nystagmus in primary position (Tables 2, 3). Perhaps the closer eye contact between younger children and their parents plays a role in the explanation of this. However, the general low prevalence of self-observed nystagmus in combination with the fact that it took more than one month from observation of nystagmus to diagnosis, indicates that there exists a general lack of awareness of nystagmus also when it occurs in primary position. We do not know if the delay is caused by patients/parents not seeking medical attention or lack of knowledge in the primary care sector. However, based on the results of the HeadSmart campaign a corresponding campaign in other countries has the potential to reduce the diagnostic interval by also informing non-professionals of common tumour symptoms including nystagmus.

We found diplopia, strabismus, other oculomotor abnormalities, and papilledema as the most common coexisting ophthalmic symptoms and signs. This is consistent with other studies investigating ophthalmic manifestations of paediatric brain tumours [8, 11]. None of the children described classic oscillopsia (visual disturbance where objects seem to oscillate/jump/vibrate), however, 16.7% reported blurred vision and hidden in that number may be oscillopsia as they are described by a child. Oscillopsia are important to help differentiate infantile nystagmus from acquired as this symptom is not present in infantile forms [17]. According to the prevalence of these symptoms and signs (Table 3), the importance of an ophthalmological examination early in the diagnostic process is emphasized. Furthermore, our study confirms the well-known dominance of symptoms/signs of increased intracranial pressure (headache, nausea, vomiting, papilledema etc.) in children with brain tumour [4, 6, 18] (Tables 3, 4).

Headache was found to be the only non-ophthalmic symptom that was age independent as underrepresented in the youngest age group (Table 4). In infants, prior to closure of the fontanels an increase in intracranial volume will cause increased head circumference and perhaps headache is not present and even if it is infants cannot verbalize their symptoms.

We found gaze-evoked nystagmus to be associated with an infratentorial tumour. This is not surprising, as tumours in this location can cause gaze-evoked nystagmus due to a deficit in the neural integrator system. The neural integrator of horizontal gaze system is mainly located in the brainstem and cerebellum and an impairment of the system will prevent maintaining the eyes in an eccentric position of gaze, leading to gaze-evoked nystagmus [12]. However, in eight cases of gaze-evoked nystagmus, the tumour was located supratentorial. This emphasizes the complexity of the pathways causing nystagmus and that tumours of several locations can affect the pathways. Also, we must have end-point nystagmus in mind, which is physiological ocular oscillations during maximal eccentric gaze occurring in many healthy subjects [13]. End-point nystagmus may be difficult to distinguish from gaze-evoked nystagmus and in the cases where the nystagmus was only observed by non-ophthalmologists, there may be a risk that some of these cases were misjudged.

The most common direction of nystagmus was horizontal (11/60) followed by a combination of different directions (9/60). In two children nystagmus was triggered by convergence. The children had tumours located in the pineal gland (Table 1) confirming their nystagmus as likely part of Parinaud’s syndrome (convergence retraction nystagmus, up gaze palsy, upper lid retraction, and pupils with light-near dissociation) – a well-known clinical sign of lesions in the dorsal mid-brain area [17]. Three children had purely torsional nystagmus indicating a lesion in the brainstem.

Unfortunately, specific descriptions of the nystagmus were missing in 60%. Hidden in this group may be other forms of nystagmus and abnormal eye movements including ocular flutter (bursts of rapid conjugate horizontal saccades without an intersaccadic interval) and opsoclonus (ocular flutter but with bursts in all directions) both of which may be associated with CNS lesions. One should keep in mind that opsoclonus and ocular flutter are also associated with neuroblastoma and children with these symptoms needs further investigations if the MRI is normal [17].

In our study more tumours were located infratentorial (70%) than supratentorial (30%), with the majority located in cerebellum and the brainstem. This is not surprising as these areas are essential in the pathways leading to nystagmus, in addition to being common sites for paediatric CNS-tumours [4, 6, 19]. Nystagmus is most commonly observed in patients with infratentorial tumours [8, 18] but supratentorial tumours may also cause nystagmus if they affect the visual pathways particularly in young children [10, 20]. In the present study tumours affecting the visual pathway were only observed in the youngest children ( < 5 years) Table 1). Optic pathway gliomas can occur in other age groups, but our study confirms that development of nystagmus caused by optic pathways gliomas are age-dependent and mainly seen in the youngest children ( < 5 years) [20, 21].

The five-year overall survival after diagnosis of the children with nystagmus was 69%. A Danish study investigating survival of a non-selected group of children with CNS tumours from 1997–2019 found a five-year overall survival at 77.6% [22]. The results are quite similar, but the groups are not fully comparable, and no definite conclusion of the mortality may be drawn.

### Limitations

The study is retrospective and based on information from medical files and thereby limited by the extent of documented information. Symptoms and/or clinical signs could have been present but not identified at the clinical examination or perhaps not documented. Detailed descriptions of the nystagmus characteristics were in many cases lacking. Not all children went through a thorough ophthalmological examination, and subtle forms of nystagmus may be overlooked, and other forms of abnormal eye movements could be mistaken for nystagmus (e.g., end-point nystagmus, opsoclonus, or ocular flutter). In the cases were the patients or parents had not noticed the abnormal eye movements we do not known when in the disease course the symptom began. Neither can we in cases where the tumour locations and the pathogenesis of the nystagmus was unclear, be sure that what was observed was not congenital and just not discovered until tumour diagnosis. The prevalence of nystagmus in the background population is unknown and any comparison with the prevalence in the children with brain tumours could not be performed.

## Conclusion

Nystagmus is frequently reported as a symptom or clinical finding in children with brain tumours. It is most often binocular, gaze-evoked, and found in combination with other complaints/clinical signs. However, it is often not discovered until the ophthalmic examination late in the time course. A simple test of oculomotor function will reveal the nystagmus and has potential to speed up the diagnostic process. Consequently, increasing awareness of nystagmus holds the potential to facilitate faster diagnosis for a subgroup of children with brain tumours.

## Summary

### What was known before

- Acquired nystagmus is an alarming symptom that may be caused by a brain tumour.
- Diagnosing children with brain tumours can be difficult because the symptoms may mimic other more common conditions.
- Children with brain tumours are prone to diagnostic delay.

### What this study adds

- Nystagmus is present in 13,7 % of children with brain tumours at the time of diagnosis.
- The majority of children (83%) have not noticed the nystagmus and the nystagmus is only identified at the ophthalmological examination late in the disease course.
- Focus on nystagmus may lead to a faster diagnosis for some children.

## Data Availability

The study was approved by the Danish Patient Safety Authority (3-3013-2800/1) and the Danish Data Protection Agency (VD-2019-84). The study was evaluated by the Regional Research Ethical Committee of the Capitol Region, Copenhagen, Denmark and deemed not to require ethics approval. The study adhered to the Declaration of Helsinki.
